# Validation of SNP Markers for Diversity Analysis, Quality Control, and Trait Selection in a Biofortified Cassava Population

**DOI:** 10.3390/plants13162328

**Published:** 2024-08-21

**Authors:** Edwige Gaby Nkouaya Mbanjo, Adebukola Ogungbesan, Afolabi Agbona, Patrick Akpotuzor, Seyi Toyinbo, Peter Iluebbey, Ismail Yusuf Rabbi, Prasad Peteti, Sharon A. Wages, Joanna Norton, Xiaofei Zhang, Adriana Bohórquez-Chaux, Hapson Mushoriwa, Chiedozie Egesi, Peter Kulakow, Elizabeth Parkes

**Affiliations:** 1 International Institute of Tropical Agriculture (IITA), Ibadan 200001, Nigeriap.kulakow@cgiar.org (P.K.); 2 Texas A&M Agrilife Research & Extension Center, Weslaco, TX 78596, USA; 3 College of Tropical Agriculture and Human Resources (CTAHR), University of Hawaii at Manoa, Hilo, HI 96720, USA; 4 Cassava Program, International Center for Tropical Agriculture (CIAT), CGIAR, Cali 763537, Colombia; 5 Department of Plant Sciences, University of California, Davis, CA 95616, USA; 6 National Root Crops Research Institute (NRCRI), Umudike, Umuahia 440001, Nigeria; 7 Plant Breeding and Genetics Section, School of Integrative Plant Science, Cornell University, Ithaca, NY 14853, USA; 8 IITA—Zambia, Southern Africa Research and Administration Hub (SARAH), Plot 1458B, Ngwerere Road (off Great North Road), Chongwe 10100, Lusaka, Zambia

**Keywords:** cassava, biofortification, population structure, KASP, DArTseq, markers, IITA, breeding

## Abstract

A validated marker system is crucial to running an effective genomics-assisted breeding program. We used 36 Kompetitive Allele-Specific PCR (KASP) markers to genotype 376 clones from the biofortified cassava pipeline, and fingerprinted 93 of these clones with DArTseq markers to characterize breeding materials and evaluate their relationships. The discriminating ability of the 36-quality control (QC) KASP and 6602 DArTseq markers was assessed using 92 clones genotyped in both assays. In addition, trait-specific markers were used to determine the presence or absence of target genomic regions. Hierarchical clustering identified two major groups, and the clusters were consistent with the breeding program origins. There was moderate genetic differentiation and a low degree of variation between the identified groups. The general structure of the population was similar using both assays. Nevertheless, KASP markers had poor resolution when it came to differentiating the genotypes by seed sources and overestimated the prevalence of duplicates. The trait-linked markers did not achieve optimal performance as all markers displayed variable levels of false positive and/or false negative. These findings represent the initial step in the application of genomics-assisted breeding for the biofortified cassava pipeline, and will guide the use of genomic selection in the future.

## 1. Introduction

The diet and caloric requirements of about 800 million people worldwide revolve around cassava, a crucial food security crop and a significant cash crop in some regions [1,2]. Although cassava roots are starch-rich, they lack critical micronutrients. Several studies have linked high cassava consumption to inadequate micronutrient intake, posing a serious public health issue that hinders sustainable development due to substantial social and economic costs [3,4,5,6,7]. One strategy that has been utilized to supply the essential nutrients required for optimum health is biofortification, a process of enhancing the nutrient density of food crops [8,9].

Biofortified cassava cultivars are a good source of provitamin A and could contribute about 40% of the overall daily intake requirement of vitamin A [10]. Biofortification has been undertaken in cassava through the HarvestPlus program (https://www.harvestplus.org/, accessed on 2 July 2024) using two main approaches, namely, transgenics and conventional breeding. The latter approach is more acceptable to the general public with demonstrated success, and several biofortified cassava varieties have been released (https://www.iita.org/news-item/nigeria-releases-cassava-higher-pro-vitamin-fight-micronutrient-deficiency/, accessed on 2 July 2024). For nutrient-improved cassava varieties to be widely accepted and adopted, the developed cultivars must also satisfy other essential qualities desired by end-users, such as meeting food quality preferences, disease resistance and/or tolerance, high dry matter content, and high yield, among others. Cassava productivity is quite limited by biotic constraints such as pests and diseases [11], notably cassava mosaic disease (CMD), which is one of the most widespread and serious diseases throughout cassava-growing areas in Africa and, more recently, in Southeast Asia, where it is an important limiting factor for cassava producers [12,13,14]. The yield loss due to CMD has been estimated to be between 45 and 97% [15].

Breeders have been under constant pressure to improve production and develop varieties that are superior in every aspect to previous varieties [16]. The biofortified breeding pipeline, in contrast to other cassava breeding pipelines at the International Institute of Tropical Agriculture (IITA), has relied on conventional breeding using a recurrent selection scheme. The recurrent selection strategy is costly, lengthy, and requires several generation and cycling evaluations. Integration of innovative approaches, like molecular markers, into conventional breeding could be a game changer. Molecular markers have a critical role to play in crop improvement and the potential to boost breeding efficiency and genetic gain. Single nucleotide polymorphisms (SNPs) are currently the marker of choice in molecular breeding and genetics due to their widespread distribution within the genome. Their wide adoption has been facilitated by the development of next-generation sequencing and various array-based genotyping platforms. Among the genotyping platforms in use are fixed array-based SNP platforms. Their main drawbacks are their high price and lack of flexibility [17,18]. Flexible technologies have been developed as an alternative. They are easy to use, cost-effective, and offer options to mix and match different SNPs [19]. Amongst the existing flexible high-throughput SNP systems currently available (TaqMan^®^, Array TapeTM, OpenArray^®^, Dynamic Arrays^TM^, iPLEX system, Target amplicon sequencing, and KASP^TM^), KASP^TM^ has grown in popularity [18]. In several crop species, including rice [20] and cowpea [21], low sets of optimal SNPs have been successfully converted into KASP assays and have served various purposes. The KASP^TM^ system is cost-effective, especially when few markers are used to genotype a large number of samples [22,23]. Using GBS-derived SNP markers from genotyping 1400 different cassava accessions, a small set of 36 quality control (QC) markers has recently been developed. These markers, along with trait-specific markers derived from genome-wide association studies [24], were converted for use as KASP assays (https://excellence inbreeding.org/module3/kasp, accessed on 2 July 2024).

The present work aims to characterize breeding materials from the IITA biofortified cassava pipeline using both DArTseq and KASP markers and to evaluate their genetic relationships. We compare the discriminatory effectiveness between the two platforms. We evaluate the prediction ability of trait-linked markers for three key cassava traits, including total carotenoid content (TCC), dry matter content (DMC), and cassava mosaic disease (CMD). Integrating molecular markers into the IITA biofortified cassava pipeline will be a game changer by providing more accurate clone (hereafter referred to as genotypes) characterization, facilitating trait prediction and enabling more precise breeding material selection; all of which will boost the speed of product development.

## 2. Results

### 2.1. Phenotypic Data Analysis

**Figure 1:**
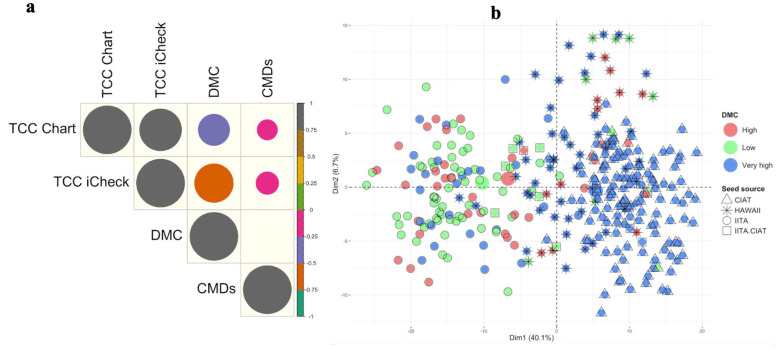

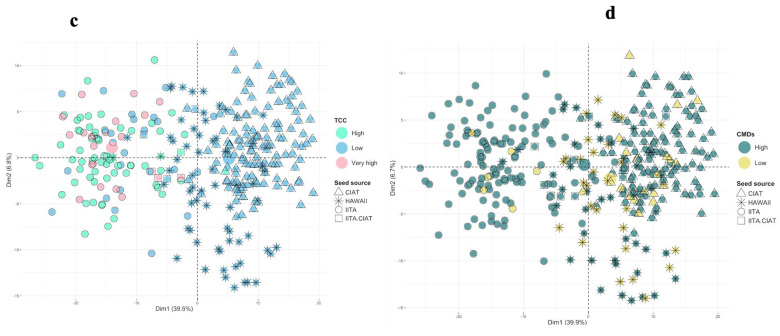
Trait correlation and the distribution of traits across the unique genotypes. (**a**) Correlation between three key cassava traits: total carotenoid content (TCC), dry matter content (DMC), and cassava mosaic disease severity (CMDs). The color scale on the left shows correlation values from +1 to −1. Grey indicates a strong positive correlation, while pink, purple, and orange represent weak, moderate, and strong negative correlations, respectively. Insignificant correlations between CMDs and DMC are shown as blank. Distribution of (**b**) DMC, (**c**) TCC, and (**d**) CMD severity across unique genotypes. Symbols represent clone sources, while colors indicate different levels for each trait. For DMC: low (<30%), high (30–33%), and very high (>33%); for TCC: low (<15 μg/g), high (15–19 μg/g), and very high (>19 μg/g); for CMD: low (<2) and high (>2).

The carotenoid concentration (TCC iCheck) was quantified using iCheck^TM^ Carotene (BioAnalyt, Berlin, Germany), a portable photometer, while the yellowness of the root parenchyma (TCC Chart) was evaluated visually using a 1 to 8 scale. The oven drying method was used to determine the percentage of dry matter content in the roots. CMD severity was assessed on a 1 to 5 scale. The Shapiro–Wilk test showed a substantial departure from a normal distribution (*p* < 0.05) for the TCC Chart, TCC iCheck, and CMDs, while DMC showed a normal distribution (*p* > 0.05) (Figure S1). All traits showed moderate or large variations among clones (Table S1). The least dispersed trait was DMC (CV = 0.2), and the most dispersed traits were TCC iCheck (CV = 0.49), followed by CMD (CV = 0.45). Correlation between traits revealed a highly positive significant correlation (r = 0.75, *p* < 0.05) between the TCC Chart and TCC iCheck. A moderate and significant negative correlation was observed between the TCC Chart and DMC (r = −0.43, *p* < 0.05). The correlation between all quantitative traits and CMDs was weak (r < 0.29, *p* < 0.05). The DMC and CMDs did not correlate (*p* > 0.05) (Figure 1a). Most genotypes (90.85%) with parental seeds from the International Centre for Tropical Agriculture (CIAT) (142) showed a high dry matter content, while genotypes with parental seeds from the International Institute of Tropical Agriculture (IITA) (120) displayed a wider range (Figure 1b). Genotypes obtained from parental seeds from the IITA biofortified cassava breeding pipeline exhibited TCC ranging from low to very high, while genotypes originating from seed sourced from the University of Hawaii (these are either first-generation crosses between CIAT and IITA source material or open-pollination seeds involving only those two source materials) and the CIAT cassava breeding program had TCC that were primarily low (Figure 1c). Regardless of the source of the seed, resistance and susceptible genotypes were present across all programs (Figure 1d).

### 2.2. Diversity Analyses Using DArTseq-Based SNP and Low-Density QC KASP^TM^ Markers

#### 2.2.1. Population Structure Analysis

**Figure 2:**
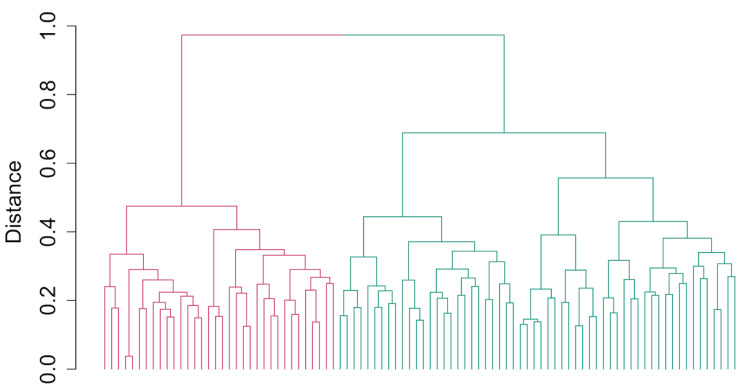
Cluster analysis showing the relationship among the 92 cassava genotypes. The 92 genotypes are separated into two clusters, with 34 and 58 accessions in clusters 1 (red) and cluster 2 (green), respectively.

**Figure 3:**
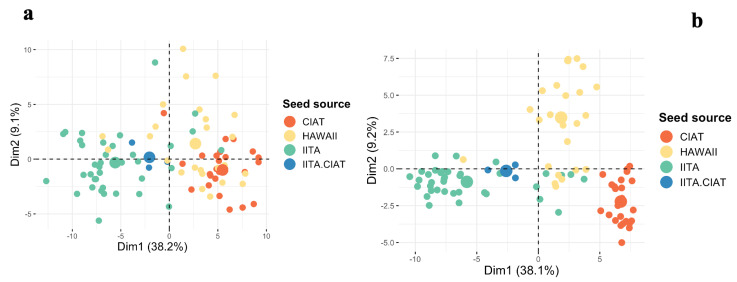
Principal component analysis obtained using the 6602 DArTseq and 36 QC KASP markers assessed in the 92 genotypes shared by both assays. The additive relationship matrix was calculated using the R package rrBLUP [25] (**a**) PCA classification using the 36 QC KASP markers; (**b**) PCA classification using the 6602 DArTseq markers. Samples are colored based on the breeding program from which the samples originated.

**Figure 4:**
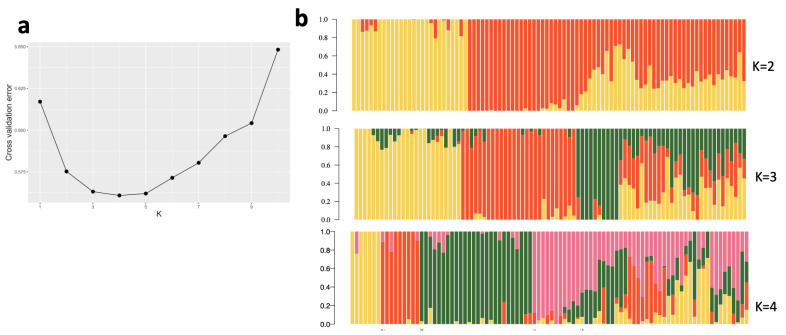
Population structure within the 92 cassava genotypes assessed with 6602 DArTseq markers. (**a**) Cross-validation plot. Cross-validation error is shown on the *Y*–axis (vertical) and the number of hypothetical populations on the *X*–axis (horizontal). Cross-validation revealed K = 4 as the optimal number of clusters. (**b**) Individual ancestry inferred with admixture. Each genotype is represented by a vertical line partitioned by color. The proportion of the color making up each vertical line represents the proportion contributed by the ancestral population. The best-supported clustering (K = 4) divided the 92 cassava genotypes into four main groups.

A total of 92 clones were genotyped using DArTseq and KASP markers. After filtering the 12,981 high-quality DArT SNP markers, 6602 markers were kept for analysis, and no sample was discarded. The dendrogram from hierarchical clustering identified two main clusters according to the elbow, silhouette, and gap statistics methods used to define the optimal number of clusters (Figure 2 and Figure S2). Cluster 1 consisted of 58 genotypes, with 26 genotypes with seed sourced from CIAT, 25 genotypes from Hawaii, and seven genotypes from IITA. Cluster 2 was composed of 30 IITA genotypes, one genotype with seed sourced from Hawaii, and three genotypes that are hybrids of IITA and CIAT genotypes. A summary of each cluster is given in Table S2. Two major groups were observed using the distance-based method and the QC KASP markers. Out of the 84 genotypes that could be compared, the estimated level of dissimilarity in terms of genotype clustering was 8.33% (Table S2). One common set of putative duplicates (IITA-TMS-IBA183098 and IITA-TMS-IBA183097) was identified using both assays, while the 36 QC KASP markers identified an additional set of putative duplicates (IITA-TMS-IBA210666 and IITA-TMS-IBA210667). Principal component analysis substantiated phylogenetic analyses with two main clusters identified (Figure S3). The genotypes were categorized according to the breeding program from which each genotype originated. Breeding materials from CIAT (seed sourced from CIAT) and Hawaii (seed sourced from Hawaii, where intercontinental hybridization is conducted to facilitate germplasm exchange) could be distinguished from one another with clear delineation using DArTseq markers, but the 36 QC KASP markers failed to clearly separate the genotypes derived from seeds sourced from Hawaii and CIAT (Figure 3 and Figure S4). A small number of Hawaii breeding materials were grouped with IITA breeding materials and vice versa (Figure 3). Admixture analysis of the 92 cassava genotypes evaluated using KASP markers supported two ancestral populations (K = 2) (Figure S5). DArTseq markers were more effective at revealing the pattern of population structure with additional detected stratification. The lowest cross-validation error identified at K = 4 suggested four ancestral populations (Figure 4).

#### 2.2.2. Genetic Diversity

There was a modest amount of genetic diversity within each of the two main clusters obtained with DArTseq (Cluster1: He = 0.33; Cluster2: He = 0.37). Both observed and expected heterozygosity were low (Cluster1: H_o_ = 0.29; Cluster2: H_o_ = 0.26). The total H_o_ considering all genotypes was lower (H_o_ = 0.28) than the total He, which was predicted to be 0.37. A similar finding was made when the genotypes were classified into groups based on the breeding program the seeds originated from. The genotypes from Hawaii showed a somewhat higher level of variation (He = 0.36) in contrast to CIAT (H_e_ = 0.35) and IITA (H_e_ = 0.34). A comparable pattern for allele richness was seen between these groups. The inbreeding (Fis) values ranged from 0.13 to 0.25 (Table 1).

#### 2.2.3. AMOVA and Population Differentiation

AMOVA analysis of the 92 cassava samples genotyped using DArTseq indicated that 8.50% of the total genetic variation occurred between the two major clusters, whereas 19.44% of the total genetic variation was found within each group. The variation within the genotypes was the highest (72.06%). Most of the molecular variance was also found to be within genotypes (73.29%) when they were categorized based on the breeding program from which the seeds were sourced, and very little variation was found between breeding programs (8.30%). Within breeding programs, only 18.71% of the total variation was seen between genotypes (Table 2). Genetic differentiation using DArTseq in the 92 genotypes was moderated between the two main clusters (*F*_ST_ = 0.09) as well as when the origin of the breeding material was considered (*F*_ST_ value ranged from 0.07 to 0.12; CIAT—Hawaii, *F*_ST_ = 0.07, CIAT—IITA, *F*_ST_ = 0.12, Hawaii—IITA, *F*_ST_ = 0.07). The *F*_ST_ between the two main groups obtained using QC KASP markers was slightly higher but still moderate (*F*_ST_ = 0.11), substantiating AMOVA’s finding of less genetic variation between clusters. These results demonstrated that low-density markers could depict genetic relationships among genotypes.

### 2.3. Evaluation of the Breeding Material Using Low-Density QC KASP^TM^ Markers

All 376 cassava genotypes were evaluated using the 36 QC markers. No markers were excluded during the filtering process, but 19 genotypes were discarded due to the low call rate (call rate < 0.85). Three hundred and fifty-seven (357) genotypes were retained for downstream analyses. Twelve pairs of putative duplicates were identified (Table S3) and 345 unique genotypes were retained for further analysis. The distinct genotypes had genetic distances ranging from 0 to 0.64. The duplicate genotypes included CIAT genotypes from different pedigrees (IITA-TMS-IBA210720 and IITA-TMS-IBA210696; IITA-TMS-IBA210741 and IITA-TMS-IBA210738; IITA-TMS-IBA210645 and IITA-TMS-IBA210649) and occasionally genotypes from the same pedigree (IITA-TMS-IBA210666 and IITA-TMS-IBA210667; IITA-TMS-IBA210757 and IITA-TMS-IBA210756). Genotypes from Hawaii were found to have duplicates (IITA-TMS-IBA210817 and IBA210816). IITA-TMS-IBA180210, a genotype used as crossing parent in IITA, turned out to be a duplicate of IITA-TMS-IBA070593 that came from Hawaii. Duplicates were found within the IITA breeding material between the elite material preserved in the field (IITA-TMS-IBA183098 and IITA-TMS-IBA18097) and the material used for crossing (IITA-TMS-IBA182959, IITA-TMS-IBA182962). IITA-TMS-IBA011368, a genotype used as a parent for crosses sampled in Ibadan, was revealed to be a putative duplicate of IITA-TMS-IBA141098, a genotype sampled from the Ubiaja genetic gain accessions, even though both genotypes displayed distinct pedigrees.

The 345 genotypes were separated into two main clusters, as shown by the dendrogram generated by hierarchical clustering, with some distinct substructures discernible within the two groups (Figure S6). PCA analysis failed to clearly separate CIAT and Hawaii genotypes, which appeared to be in the same cluster, from IITA breeding material (Figure S7). This information was corroborated by the low genetic differentiation between genotypes from Hawaii and CIAT (*F*_ST_ = 0.05), as well as the higher genetic differentiation between breeding material from CIAT and IITA (*F*_ST_ = 0.11). Moderate genetic differentiation was reported between Hawaii and IITA breeding material (*F*_ST_ = 0.07). AMOVA analysis revealed low genetic variation between groups compared to within-group variation (Table S4).

### 2.4. Marker-Assisted Trait Selection

The trait-linked markers used in this study derived from genome-wide association studies. The favorable allele at each trait locus was determined by assessing the allele substitution effect [24]. The SNPs were converted into KASP markers (https://excellenceinbreeding.org/module3/kasp, accessed on 2 July 2024).

#### 2.4.1. Marker Segregation and Marker Effects on the Traits

##### Cassava Mosaic Disease

**Figure 5:**
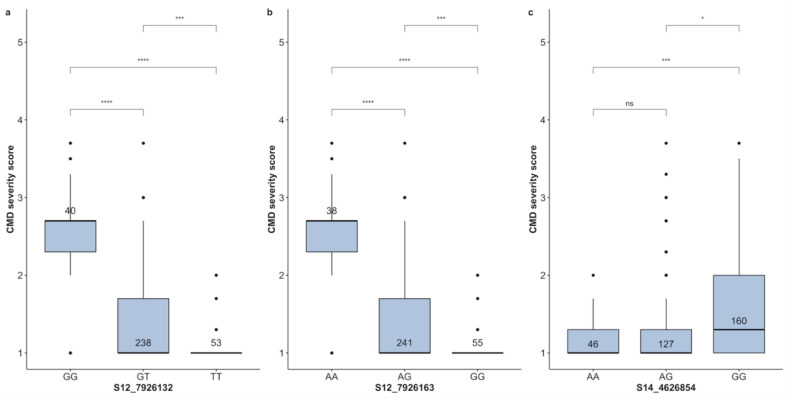
Boxplot showing allelic substitution effect for cassava mosaic disease severity. Three markers, namely S12-7926132 (**a**), S12-7926163 (**b**), and S14-4626854 (**c**), were evaluated. The genotypes/alleles of each marker are presented on the *X*–axis, while the phenotype trait and its value are mentioned on the *Y*–axis. The Kruskal–Wallis test was used to assess the statistical differences between genotype classes. The stars in the figure indicate levels of statistical significance. *, ***, **** significant at *p* ≤ 0.05, *p* ≤ 0.001, *p* ≤ 0.0001, respectively; ns = non-significant (*p* > 0.05).

Three markers, including S12-7926132, S12-7926163, and S14-4626854 [24] were used to evaluate the 331 clones with both phenotypic and genotypic data (Table 3). At marker S12-7926132, the CMD-linked markers, the frequency of homozygote for the favorable allele (T) was estimated at 16.01%, which was also the major allele. In contrast, the highest frequency for clones was for heterozygotes (71.9%), and 12.8% of genotypes were homozygotes for the unfavorable allele (Table 3). At marker S12-7926163, the unfavorable allele (A) was detected in a homozygote state at a frequency of 11.38%, whereas the favorable alleles were present in 88.63% of the genotypes, either in a homozygous (16.47%) or heterozygous (72.16%) state. Markers S12-796132 and S12-7926163 displayed a pattern of segregation typical of dominant markers. For both markers, a significant difference was observed among the three genotypic classes. The heterozygote genotypes displayed different levels of resistance (Figure 5a,b). Among the 333 clones with genotypic and phenotypic data at marker S14-4626854, the favorable allele (A) was found in approximately half of the 333 clones (51.95%), either in a homozygote state (13.81%) or heterozygote state (38.14%), while the remaining genotypes were homozygous for the unfavorable allele (48.05%) (Table 3). Significant CMD resistance was displayed by the 46 homozygous clones for the favorable alleles (CMDs score ≤ 2.0). S14-4626854, a marker on chromosome 14, showed ambiguous discriminatory power (Figure 5c). Indeed, the homozygous genotype for the favorable allele (AA) and the heterozygous allele (AG) did not differ significantly and showed low scores of CMD compared to genotypes with unfavorable alleles (GG). It was recently shown that this marker had been misplaced in previous genome assembly and is, indeed, located on chromosome 12 linked to other markers.

##### Dry Matter Content 

**Figure 6:**
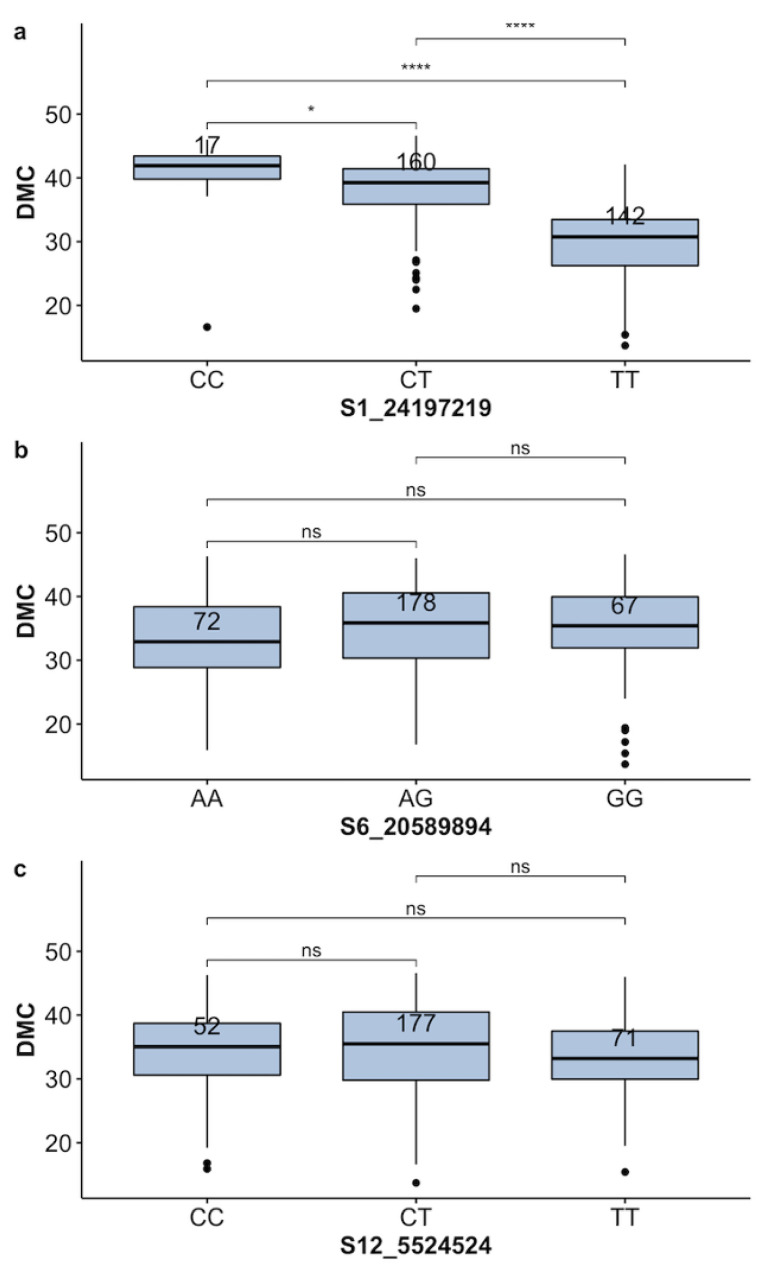
Boxplot showing allelic substitution effect for root dry matter content. Three markers, namely S1-24197219 (**a**), S6-20589894 (**b**), and S12-5524524 (**c**), were evaluated. The genotypes/alleles of each marker are presented on the *X*–axis, while the phenotype trait and its value are mentioned on the *Y*–axis. The Kruskal–Wallis test was used to assess the statistical differences between genotype classes. The stars in the figure indicate levels of statistical significance. *, **** significant at *p* ≤ 0.05 and *p* ≤ 0.0001, respectively; ns = non-significant (*p* > 0.05).

Three markers, including S1-24197219, S6-20589894, S12-5524524 [24] were used to evaluate the 319 clones with genotypic and phenotypic data. At marker S1-24197219, 5.33% of the clones were homozygous for the favorable allele (C) and had high dry matter content. Half of the clones (50.16%) had one copy of the favorable allele, whereas 44.51% contained the unfavorable allele (Table 3). The allele substitution effect revealed an additive segregation pattern for the marker S1-24197219, with no significant differences between genotypes carrying homozygous favorable alleles with heterozygous genotypes (Figure 6a). Favorable alleles (G) at marker S6-2058894 were found in 77.29% of the 317 clones with phenotypic and genotypic information, whereas 22.14% were homozygous for the unfavorable allele. The favorable allele (C) linked with high DM at marker S12-5524524 was estimated to be 15.32% and 55.2% in homozygote and heterozygote states, respectively, while only 23.7% of the total genotypes carried the unfavorable allele. The segregation pattern of marker S6-20589894 was inconsistent with the phenotypic distribution of the two homozygous states (Figure 6b). Similar results were obtained for marker S12-5524524, which also displayed an erratic pattern of segregation. Neither marker S6-20589894 nor marker S12-5524524 displayed significant differences between genotypic classes (Figure 6c).

##### Provitamin A

**Figure 7:**
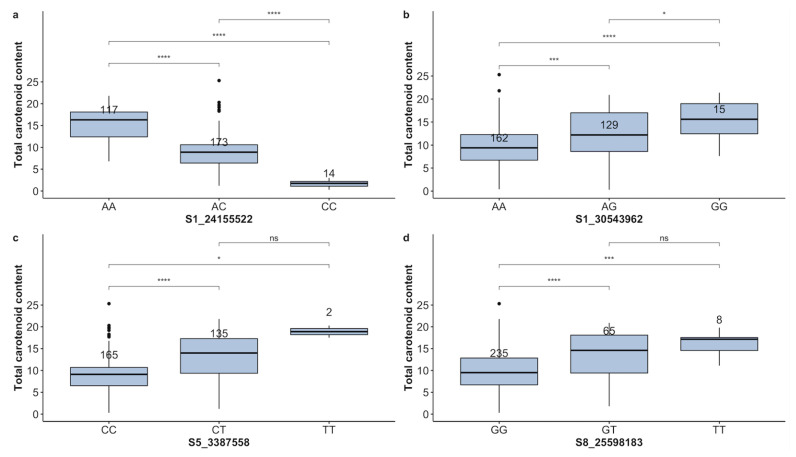
Boxplot showing allelic substitution effect for total content of carotenoids, estimated using iCheck. Four markers, namely S1-24155522 (**a**), S1-30543962 (**b**), S5-3387558 (**c**), and S8-25598183 (**d**), were evaluated. The genotypes/alleles of each marker are presented on the *X*–axis, while the phenotype trait and its value are mentioned on the *Y*–axis. The Kruskal–Wallis test was used to assess the statistical differences between genotype classes. The stars in the figure indicate levels of statistical significance. *, ***, **** significant at *p* ≤ 0.05, *p* ≤ 0.001, *p* ≤ 0.0001, respectively; ns = non-significant (*p* > 0.05).

Four markers, including S1-24155522, S1-30543962, S5-3387558, and S8-25598183 [24] were used to evaluate the clones. When TCC (Provitamin A) was assessed using the ICheck^TM^ device, among the clones with both phenotypic and genotypic data at marker S1-24155522, the favorable allele (A), which was also the major allele, was present in 95.4% of the clones in either the homozygous state (38.49%) or the heterozygous state (56.91%), demonstrating that this beneficial allele is widespread within the clones and has been brought to fixation in the breeding pipeline. A few genotypes were homozygous for the unfavorable allele (Table 3). The TCC level was higher in genotypes homozygous for the favorable allele than in heterozygous genotypes (Figure 7). Significant differences between marker classes and an additive allelic effect were identified for marker S1-24155522 (Figure 7a). For the markers S1-30543962, S5-3387558, and S8-25598183, all minor alleles were reported as favorable alleles with a frequency ranging from 0.66 to 4.90% in the homozygous state. The remaining clones that carried the favorable allele were in the heterozygote state (21.10–44.70%) (Table 3). These markers exhibited dominant gene effects as genotypes with at least one copy of the favorable allele displayed high levels of TCC (Figure 7b–d). When the Tchart was used to assess the TCC content, markers S5-3387558 and S8-25598183 showed a dominant allelic effect, marker S1-24155522 displayed an additive allelic effect, and marker S1-30543962 did not show a segregation pattern consistent with the expression of the trait. There was no statistically significant difference between the co-segregating phenotypes of the two homozygous allelic states for each of the three markers. However, the homozygous genotype for the favorable allele co-segregates with high TCC content (Figure S8).

#### 2.4.2. Marker Quality Metrics

All three CMD-linked markers had a high rate of false positives (26.42–44.23%). For markers S12-7926132 and S12-7926163, the chance of incorrectly scoring each genotype as QTL− was low (3.28% and 3.23%, respectively), while marker S14_4626854 showed a high proportion of false negatives (43.32%) (Table 3). As for DMC trait-linked markers, the risk of misclassifying genotypes either as QTL−, QTL+, or both, and advancing/discarding genotypes based on marker information, was large (17.33–75.00%) (Table 3). No ideal score (0%) was obtained when classifying genotypes as white or yellow roots. Furthermore, a high proportion of genotypes were reported as false positives (19.57–55.88%), false negatives (0–73.99%), or both.

## 3. Discussion

The collaboration between IITA, CIAT, and the University of Hawaii has facilitated the exchange of advanced elite clones and germplasm, bringing the value of Latin American germplasm and expanding the genetic base of cassava in Africa [26]. CMD resistance genotypes were observed throughout the genotypes from the three programs analyzed, translating the introgression of CMD-resistant alleles in African and Latin American germplasms and the widespread use of CMD-resistant clones or varieties as parents and CMD donors [26]. A broad range of total carotenoid content was found in the breeding materials of IITA’s biofortified cassava pipeline. This is in line with the pipeline’s primary goal of increasing TCC, and translates the presence of adequate genetic variability among cassava genotypes and the complex interactions among the parental genotypes [27]. The requirement for combining high TCC with high DMC, a key determinant of product acceptance by farmers and processors, is now recognized. CIAT has generated a number of elite clones with superior and stable DMC, and other high-value traits, some of which have been introduced into IITA cassava breeding programs. The high DMC of genotypes derived from CIAT parental seeds corroborates the purpose of the crosses, which was to improve the culinary quality of the developed genotypes. A challenge to concurrently improve both traits is the negative association between DMC and TCC, which has also been documented in previous studies [27,28]. The strong correlation between the root TCC Chart and TCC ICheck suggests that visual evaluation could be used effectively as a proxy to indirectly select genotypes with adequate TCC levels. This would speed up the evaluation and advancement of genotypes, especially in the early stages of product development (e.g., seedling nursery) when a large number of accessions need to be assessed. The low correlation between TCC and CMDs, or between DM and CMDs, indicates that these traits are independently inherited and could be improved concomitantly. The breeding materials under evaluation displayed considerable phenotypic variation for all traits and could be a reservoir from which promising clones serving as parents would be selected. Realigning the breeding strategy in the IITA biofortified breeding pipeline with the application of genomic tools could add an additional layer of information, facilitate parents’ selection, and indubitably address some persistent challenges, including combining yield, dry matter, and quality in biofortified lines.

For the first time, the genetic relatedness between selected genotypes that make up the breeding material for the biofortified pipeline at IITA was assessed using SNP markers. The SNP markers efficiently classified the genotypes into two main groups. Genotype clustering was consistent with pedigree information and reflected the various ancestors that make up the genetic materials. Genotypes’ separation was connected to the breeding program the seed came from, and substantial divergence between materials from various programs was highlighted. The distinctiveness between genotypes from various origins might translate into the presence of specific alleles within each program. Classification of breeding materials into genetic groups is crucial as it guides the selection of parents and reduces mating between related genotypes. Such analysis could be useful for planning crosses and optimizing hybridization efficiency, which could result in the generation of highly phenotypic contrasting progenies for various traits [29,30].

There was a moderate level of variation and differentiation between the groups, which was likely caused by (1) the selection of similar traits by CIAT and IITA breeding programs; (2) introgression that might have occurred between genotypes as revealed by the pedigree information, which showed a considerable number of genotypes sharing common parents; and (3) germplasm movement between the three programs (CIAT, Hawaii, and IITA). Indeed, crossing efforts to combine complementing traits have been conducted between African and Latin American breeding materials. As a result, many IITA breeding materials do have a CIAT background. The materials from Hawaii represent first-generation hybrids between CIAT and IITA breeding materials, which may also explain the lower differentiation observed. Most of the variation was observed within genotypes, which is consistent with findings made by Oyesigye et al. [31]. Cassava is highly heterozygous, thus, the moderate amount of genetic diversity reported in this study may be explained by the close relatedness among breeding materials as opposed to the high heterozygosity typically found in diverse genotypes. The slightly higher heterozygosity of Hawaii (He = 0.36) and CIAT (He = 0.35) breeding material in comparison with IITA breeding material corroborates the finding of [32], who reported a higher diversity in the CIAT breeding materials compared to the IITA breeder’s germplasm. A number of hypotheses might explain these differences: (1) CIAT is acknowledged as a center of cassava diversity and domestication of the crops, while Hawaii, a transit center to facilitate the transfer of alleles between continents, is where hybridization is conducted using CIAT and IITA seed sources; (2) the low diversity of IITA material might be related to the incorporation of specific traits with limited sources in the biofortified pipeline. As expected for an outcrossing crop, the amount of inbreeding was not consistently close to zero, suggesting a potential heterozygosity deficit, outcrossing of closely identical genotypes in close proximity in the field, or crosses between related individuals during the breeding process [32,33].

Some genotypes were flagged as putative duplicates. The occurrence of duplicate genotypes has been reported in several studies [29,34,35]. In cassava breeding programs, recurrent selection is routinely used, and many genotypes are evaluated through several stages and different environments, particularly at the early stage of the breeding process. Advanced lines are shared between partners and stems are multiplied. Leaves are sampled for various purposes. Through all these processes, errors could happen at any stage and might have severe repercussions for product development, including time wasting, resources, and reduced genetic gain [36]. Therefore, genotype identity must be verified at every stage of product development to prevent duplication. This justifies the implementation of routine quality control to enhance breeding effectiveness and enable efficient resource utilization. The genotype data alone cannot serve as ambiguous duplicate confirmation; this must be followed by the phenotypic evaluation of the potentially identified duplicate genotypes [37].

The value of the 36 cassava QC KASP markers for routine evaluation and characterization of breeding materials was highlighted in the current study. In contrast to DArTseq markers, KASP markers overestimated the prevalence of duplicates and demonstrated low resolution in differentiating genotypes based on seed sources. This is most likely due to the lower marker number, which might be less effective in separating closely identical genotypes. Nevertheless, the general structure of the population was similar using both assays. Similar findings by earlier authors demonstrated that a minimal number of high-quality markers is sufficient and that employing high-density markers adds little value. Ertiro et al. [38] compared the correlation between low-density and GBS-based high density markers, and reported that only small subsets of preselected, high-quality markers were necessary for QC analysis in maize. Gemenet et al. [20] reached the same conclusion after comparing a high-density SNP set of 10,159 markers with a select set of 30 SNPs in a sweet potato breeding program. Both sets of markers identified relatively similar mislabeling errors. Tang et al. [23] found that both sets of 331 and 48 KASP markers were equally suitable for analyses of conventional rice varieties. KASP markers are flexible, and genotyping can be tailored by selecting specific SNPs that are relevant to the study. When using a large number of genotypes with a small set of markers, KASP markers are cost-effective. Fifty markers assessed against 1536 samples are estimated to cost about $7 per sample, compared with DArTseq, which costs approximately $21 per sample. The genotyping data from KASP do not require much bioinformatic support. KASP markers will therefore be useful for quality assurance and control in breeding trials. On the other hand, DArTseq markers are valuable tools for comprehensive diversity and recommended markers for genomic selection.

We assessed the utility of individual trait-linked markers in unambiguously distinguishing genotypes with favorable alleles. All markers displayed variable levels of false positive and/or false negative, and none of them achieved the ideal rate of zero percent reported in some recent studies [39,40]. The aforementioned observations contradict some early cassava studies that found a significant association between a number of trait-linked markers and expressed phenotypes, including markers S1-24197219 and DMC, S1-24155522, and TCC, S12_796132, and S12_796163 with CMDs [41,42,43]. These significant associations were prevalent in certain families, but when pre-breeding population was used, the results were inconsistent [41,42]. False positives and false negatives happen, pose serious concerns, and have been reported in previous studies [44]. The discrepancy between marker genotypes and the plant phenotype suggests that there is a considerable risk in advancing or discarding clones based on the trait-linked marker information and limits their broad application in breeding programs. The strong marker predictability of some of these trait markers was previously attributed to their near proximity to functional genes and/or regulatory regions [41,43]. Numerous factors, such as the genetic distance between the marker and the gene/QTL promoting genetic recombination and separating the marker from the associated QTL, could explain the disparity between genotype data and the expressed phenotype seen in the current study [36,45]. Indeed, a portion of the plant materials used are hybrids between external germplasm from Latin America and elite genotypes of the IITA biofortified breeding pipeline. Hybridization has been carried out to increase the frequency of favorable alleles and to incorporate desirable traits required for the development of new varieties. Following hybridization, recombination can disrupt the linkage between alleles at different loci, produce novel combinations of parental alleles across loci, alter haplotype structure, modify allelic association between trait-linked markers and QTLs, and make it more challenging to predict phenotypes accurately [46,47,48]. While other authors have reported that trait-linked markers’ optimal performance may be limited to specific crosses, Singh et al. [49] have shown that haplotype variation of markers not associated with the casual gene can result in falsely positive or falsely negative results [44,50]. This incongruency might well translate into a reduction in pathogen effectiveness [51], low phenotypic variation contributed by the QTLs and their minor effect, and epistatic interaction between QTLs that could inhibit the expression of beneficial alleles [52]. Another possible cause is the potential bias in the phenotypic evaluation process. The existence of other independent loci, not yet captured and underlying the traits or modifying factors, cannot be ruled out. Trait-linked markers used individually may not be predictive in an unknown background, despite some authors’ claims that the best markers can perform as well as, or better, than marker haplotype [46]. An alternative that should be investigated is using the trait-linked markers as haplotypes [40].

The evaluated breeding materials are a reservoir from which promising clones serving as parents could be selected to support ongoing population improvement. The characterized genotypes are conserved in the field, and several are at varying stages of product development. Routinely deploying KASP markers across the cassava breeding pipeline will be needed to eliminate errors associated with mislabeling before genotypes are advanced or exchanged. Another important implementation for effective use of the genotypes will be pedigree reconstruction

## 4. Materials and Methods

### 4.1. Plant Materials

The set of cassava clones (hereafter referred to as genotypes) used in this study was composed of selected genotypes from clonal evaluation trials (CETs), advanced yield trials (AYTs), genotypes frequently used as parents for crosses, and a subset of the genetic gain (GG); which is a collection of elite tropical *Manihot* selections (TMS) selected over the past five decades and landraces. The specific samples were collected from different trials established in two different locations in Nigeria: Ubiaja and Ibadan. While some of the plant materials are breeding lines from the IITA cassava biofortified breeding programs, some plant materials are high-priority clones selected from the International Centre for Tropical Agriculture (CIAT) and the University of Hawaii (Hawaii). The average trait value for each genotype was used for downstream analyses. In Table S2, the initial set of 376 genotypes used is listed.

### 4.2. Phenotyping Evaluation

Cassava mosaic disease, dry matter content, and total carotenoid content were among the phenotypic traits scored. Mature roots were harvested at 12 months after planting for assessment of TCC and DMC. The phenotypic data used are the mean value of each trait for each genotype. Detailed descriptions of the phenotyping approaches are provided below.

#### 4.2.1. Cassava Mosaic Disease Severity

The severity of cassava mosaic disease was scored at three, six, and nine months after planting on a scale of 1 to 5 [53], with 1 = no symptoms observed or mild distortion at the base of leaflets, with the remaining leaflets appearing green and healthy; 2 = mild chlorotic pattern on entirety of leaflets; 3 = strong mosaic pattern on entire leaf, and narrowing and distortion of lower one-third of leaflets; 4 = strong mosaic pattern on entire leaf, and narrowing and distortion of lower one-third of leaflets; 5 = strong mosaic pattern on entire leaf, and narrowing and distortion of lower one-third of leaflets. The average CMD severity was computed for each plant.

#### 4.2.2. Dry Matter Content Measurement

Six healthy storage roots of varying sizes (small, medium, and large) were randomly selected from each plot to ensure representativeness. Selected roots were free of defects such as decomposition, disease, and bruises. The harvested roots were placed in labeled sampling bags and immediately transported to the laboratory for root dry matter analysis. Roots were peeled, washed under running water, and blotted dry with paper towels. The proximal and distal ends of each root were cut off. A hand grater (3-mm hole diameter) was used to shred the top, middle, and bottom sides of each selected root. Following thorough mixing of all the shredded roots from each plot, 100 g of the homogenized sample was oven-dried at 80 °C for approximately 72 h. The dry samples were weighed, and root dry matter content (RDMC) was calculated as the ratio of the dry weight to the fresh weight as follows:

$$\mathrm{RDMC}\;(\%)=\frac{Dryweight}{Freshweight}\times 100$$

#### 4.2.3. Visual Evaluation of the Yellowness of the Root Parenchyma

A color chart approach [54] was used to visually assess the root parenchyma to determine the variation in carotenoid content among cassava clones, where 1, 2, 3, 4, 5, 6, 7, 8 represent white, light cream, cream, light yellow, yellow, deep yellow, orange, pink, respectively. Four healthy roots randomly selected from each plot, and the most often observed score for each genotype, were recorded.

#### 4.2.4. Icheck Carotene™

The total carotenoid content (TCC) was quantified using the protocol from Esuma et al. [27] with some modifications. Five healthy storage roots of varying sizes (small, medium, and large) were randomly selected from each plot. Selected roots were devoid of defects like decomposition, disease, and bruises. These harvested roots were placed in labeled sampling bags and immediately transported to the laboratory for TCC quantification. Once at the laboratory, roots were peeled, washed under running water, and blotted dry with paper towels. Each of the five roots was cut longitudinally into four equal pieces, with the two opposing sections chopped into cubes, homogenized, and 5 g of the homogenized root samples collected. The homogenized root sample was pounded and ground with a mortar and pestle into a smooth paste. To help grind the sample, 20 mL of distilled water was gradually added. The resulting slurry was transferred into a 50-mL calibrated falcon tube and topped up with 25 mL distilled water. The falcon tube content was shaken vigorously for 10 s, and 0.4 mL of the solution (slurry) was injected into the iExTM Carotene vial (BioAnalyt, Berlin, Germany) using the syringe and needle provided with the kit. Prior to the reading, vials were left on a stable surface for around five minutes. Two solution phases, a clear upper phase and a turbid lower phase, appeared inside the vial. Using the iCheck^TM^ Carotene equipment (BioAnalyt, Berlin, Germany), the upper phase’s absorption was measured, and the total carotenoid content in the cassava roots expressed as µg g^−1^ was obtained by multiplying the dilution factor (DF = sample weight/volume of slurry) with the device reading.

TCC (µg g^−1^) = DF × device reading

### 4.3. Phenotypic Data Analysis

Descriptive statistics for each trait were computed using the “describe” function from the R package “Pastecs” [55]. The variation in the phenotypic data sets was visualized using histograms, and the Shapiro–Wilk test was applied to check the normality of each trait. The function “rCorr” in the R package “Hmisc” [56] was used to conduct correlations between traits and determine the significance of those correlations.

### 4.4. Leaf Sampling, Genotyping, and Quality Control Analyses

Three to four leaf discs of 6 mm in diameter were collected from the young leaves of each tagged clone using a hole puncher. The leaf discs from each sample were transferred with forceps into a single well of a 96-well PCR plate (1.2 mL abGene Storage Plate, Number AB0564, Thermo Fisher Scientific, Waltham, MA, USA) with the corresponding plant ID for each sample. Forceps were cleaned before and after inserting each sample into a well to prevent cross-contamination. Plant tissue was preserved on ice for transport from the field to the laboratory. Samples were stored in a −80 °C freezer before freeze-drying in a lyophilizer at −51 °C and 5.0 pa for a minimum of 72 h. Freeze-drying was performed using a LABCONCO FreeZone 18-L, −50 °C freeze-dryer (Kansas City, MO, USA). Freeze-dried samples were shipped to the Intertek–Agritek Laboratory in Australia for total genomic DNA extraction and genotyping using a KASP^TM^ genotyping platform assay, while a subset of 93 samples was sent to Diversity Arrays Technology in Canberra, Australia, for genotyping by DArTseq technology (https://www.diversityarrays.com/, accessed on 2 July 2024). Upon receipt of the genotypic data, quality control was performed using Tassel 5 [57]. A filter was applied to remove markers with a call rate of less than 0.85 (call rate < 0.85) and/or minor allele frequency under 0.05 (MAF < 0.05). Samples with a call rate of 0.80 were retained for further analyses. Filtered KASP and DArTseq genotypic data were used for downstream analyses.

### 4.5. Diversity Analyses Using DArTseq-Based SNP Markers

A subset of 92 genotypes were randomly sampled (Figure S9) and genotyped using DArTseq. Putative duplicates were identified using an arbitrary threshold value set at 0.05 and visualized using the “heatmap” function of the base R package “stats”. The population structure, population statistics, and genetic parameters were assessed using the unique set of genotypes.

#### 4.5.1. Assessment of Population Structure

The genetic distance among the 92 selected genotypes was computed using PLINK version 1.9 [58]. The pairwise distance matrix was used to visualize relationships between genotypes. Hierarchical cluster analysis was performed using the “hclust” function and the “Ward.D2” method in the “stats” package in R. The hclust objects were converted into a tree class using the R package “ape” [59] and the phylogenetic tree was visualized using the “plot” function of the R statistical software version 4.3.2 [60]. The optimal number of clusters was determined using the elbow, silhouette, and gap statistic methods. The maximum likelihood estimate implemented in the admixture program [61] was used to assign individual genotypes to hypothetical founder populations. A tenfold cross-validation procedure was used to define the most suitable K value. The number of clusters (K) computed ranged from 1 to 10. The proportion of the putative ancestral population of each genotype was defined in the Q matrix. Genotypes were assigned to a group if the probability of their group membership determined by admixture was ≥80%. The level of genetic affinity among breeding materials was also assessed by principal components analysis using the R package “rrBLUP” [25]. PCA function in the FactoMineR package in R was used to perform principal component analysis (PCA) [62].

#### 4.5.2. Estimates of Genetic Diversity Parameters, Analyses of Molecular Variance (AMOVA), and Population Differentiation

Several metrics were estimated, including observed heterozygosity (Ho), the inbreeding coefficient for each population (F_IS_), and expected heterozygosity (He) using the R package Adegenet [63]. The allelic richness (Ar), the population-specific genetic differentiation, and population-specific *F*_ST_ bootstrap confidence interval were computed using the hierfstat R package [64]. The population differentiation was evaluated using pairwise *F*_ST_ estimates, according to [65], using the pairwise.betas function built into the hierfstat package [64], where *F*_ST_ < 0.05 indicates low differentiation; 0.05 < *F*_ST_ < 0.15 indicates moderate differentiation; and >0.15 indicates considerable genetic differentiation. Analysis of molecular variance (AMOVA) was performed to determine the partition of genetic variation among populations and/or groups. These analyses were carried out using the R package Poppr [66].

### 4.6. Comparative Analysis of DArTseq and Quality Control KASP^TM^ Markers

The discriminatory ability of the 6602 DArTseq and 36 QC markers was assessed in the 92 genotypes shared by both assays. The population structure was compared, and the degree of misclassification or difference in genotype classification between the two platforms was evaluated.

### 4.7. Evaluation of Breeding Material Using Quality Control KASP^TM^ Markers

The initial set of cassava genotypes’ genetic relationships was assessed using quality control markers. The genetic distance between the genotypes was computed using PLINK [58]. The pairwise distance matrix was used to visualize relationships among genotypes. Hierarchical cluster analysis was performed using the hclust function and the Ward.D2 method built into stats, a base R package. The hclust objects were converted into a tree class using the R package “ape” [59], and the phylogenetic tree was displayed using the plot function of the R statistical software [60]. Putative duplicates were identified using an arbitrary threshold value set at 0.05 and visualized using the “heatmap” function of R stats. The optimal number of clusters was determined using the elbow, silhouette, and gap statistic methods.

### 4.8. Marker-Assisted Trait Selection

#### 4.8.1. Marker Segregation and Marker Effects

Phenotypic distributions between different genotype classes for each trait were compared and visualized using the “ggboxplot” function built into the “ggpubr” R package [67]. The Kruskal–Wallis test was used to assess the statistical differences between genotype classes.

#### 4.8.2. Trait-Marker Quality

The false positive rate (FPR, which occurs when a marker predicts the presence of a trait (QTL+) that is not reflected in the phenotype) and the false negative rate (FNR, which occurs when a marker predicts the absence of a trait (QTL−), but the phenotype is contradictory) were the two variables used to evaluate trait-marker quality. The percentage of genotypes classified as such was quantified as proposed by Platten et al. [39], where FPR is assayed as the number of known recipients identified as not having an unfavorable allele(s) of the marker (and thus incorrectly classified as QTL[+]) and FNR is the converse, i.e., the proportion of known QTL[+] genotypes incorrectly classified as QTL[−] due to not having a favorable allele of the marker.

## 5. Conclusions

It is crucial to integrate innovative approaches throughout all cassava pipeline breeding to satisfy the rising demand for cassava food and non-food products, and enhance the density of total carotene content. We characterized IITA biofortified cassava breeding materials using medium- and low-density markers. Mainstreaming the latter in the IITA cassava biofortified breeding pipeline will be cost-effective and enable the identification of any errors or mix-ups and mislabeling that have unintentionally occurred, and their correction. This will ensure the integrity of breeding materials and the fidelity of crosses. The prevalence of false negative and positive results emphasizes the need to assess the markers against different genetic backgrounds to ensure their stability. The knowledge gained from this research work will help with the management and efficient use of the IITA biofortified cassava breeding materials.

## Figures and Tables

**Table 1:** Summary statistics of genetic diversity indicators by group and between breeding programs of origin of the 92 cassava samples genotyped using DArTseq.

	Ho	He	Fis	Ar	*F*st	CI_2.5%	CI_97.5%
K2-Cluster1	0.29	0.37	0.19	2.37	0.04	0.04	0.05
K2-Cluster2	0.26	0.33	018	2.29	0.13	0.13	0.14
92 samples (overall)	0.28	0.37	0.25		0.09	0.09	0.09
CIAT	0.29	0.35	0.13	2.18	0.09	0.08	0.096
IITA	0.27	0.34	0.20	2.31	0.10	0.09	0.11
Hawaii	0.28	0.36	0.18	2.29	0.06	0.06	0.09
89 samples (overall)	0.28	0.37	0.21		0.08	0.08	0.09

Observed heterozygosity across loci (Ho). Mean allele richness (Ar). Expected heterozygosity across loci (He). Inbreeding coefficient (Fis). Population-specific genetic differentiation (*F*_ST_), population-specific *F*_ST_ bootstrap confidence interval (CI_2.5% and CI_97.5%).

**Table 2:** Analysis of molecular variance of the 92 cassava samples genotyped using DArTseq.

	Source of Variation	Degree of Freedom	Sum of Squares	Mean Sum of Square	Estimate Variance	% of Variation
K = 2	Between cluster	1	21,438.06	21,438.064	217.01	8.50
	Between genotypes within cluster	90	254,892.12	2832.135	496.22	19.44
	Within genotypes	92	169,251.00	1839.685	1839.68	72.06
	Total	183	445,581.18	2434.870	2552.92	100.00
Seed sources	Between seed sources	2	29,880.2	14,940.10	208.40	8.30
	Between genotypes within seed sources	86	237,676.4	2763.68	462.08	18.41
	Within genotypes	89	163,717.0	1839.52	1839.52	73.29
	Total	177	431,273.6	2436.57	2510.00	100

**Table 3:** Segregation of marker genotypes for each phenotype.

Trait	Markers	Favorable Allele	Homozygous Minor Allele	Heterozygous	Homozygous Major Allele	% Homozygous Minor Allele	% Heterozygous	% Homozygous Major Allele	FPR (%)	FNR (%)
CMD	S12-7926132	T	GG	GT	TT	40	238	53	42.59	3.28
CMD	S12-7926163	G	AA	AG	GG	38	241	55	44.23	3.23
CMD	S14-4626854	A	AA	AG	GG	46	127	160	26.42	43.32
DMC	S1-24197219	C	CC	CT	TT	17	160	142	17.33	32.79
DMC	S6-20589894	G	GG	AG	AA	67	178	72	72.00	21.07
DMC	S12-5524524	C	CC	CT	TT	52	177	71	75.00	23.35
TCC	S1-24155522	A	CC	AC	AA	14	173	117	55.88	−
TCC	S1-30543962	G	GG	AG	AA	15	129	162	52.17	48.81
TCC	S5-3387558	T	TT	CT	CC	2	135	165	36.96	53.56
TCC	S8-25598183	T	TT	GT	GG	8	65	235	19.57	73.99

CMD, cassava mosaic disease; DMC, dry matter content; TCC, total carotenoid content; FPR, false positive rate; FNR, false negative rate.

## Data Availability

Data are contained within the Supplementary Materials.

## Supplementary Materials

The following supporting information can be downloaded at: https://www.mdpi.com/article/10.3390/plants13162328/s1, Table S1. Descriptive statistics of the traits. Table S2. The initial 376 cassava genotype used in the study. Table S3. Putative duplicates identified from the filter set of 357 genotypes using KASP markers. Table S4. Analysis of molecular variance of the 345 unique cassava genotypes assessed using KASP markers. Figure S1. Histogram showing the distribution of each trait. Figure S2. The optimal number of clusters defined using three approaches. Figure S3. Principal component analysis of 92 cassava genotypes genotyped with DArTseq. Figure S4. Dendogram showing the phylogenetic relationships between the 92 cassava genotypes genotyped using DArTseq. Figure S5. Population structure within the 92 cassava genotypes assessed with 36 KASP markers showing partitioning into two main populations. Figure S6. Cluster analysis showing the relationship among the 345 unique cassava genotypes. Figure S7. Principal component analysis of the 345 unique cassava genotypes assessed with the 36 QC KASP markers. Figure S8. Boxplot showing allelic substitution effect for total carotenoid content, estimated using color chart. Figure S9. Representative set of samples genotyped using DArTseq (yellow color) from the unique genotypes evaluated.
