# Supplementary figures and images for: Validation of SNP Markers for Diversity Analysis, Quality Control, and Trait Selection in a Biofortified Cassava Population
Source: Plants (Basel). 2024 Aug 21;13(16):2328. doi: 10.3390/plants13162328 (PMC11359368; doi:10.3390/plants13162328)

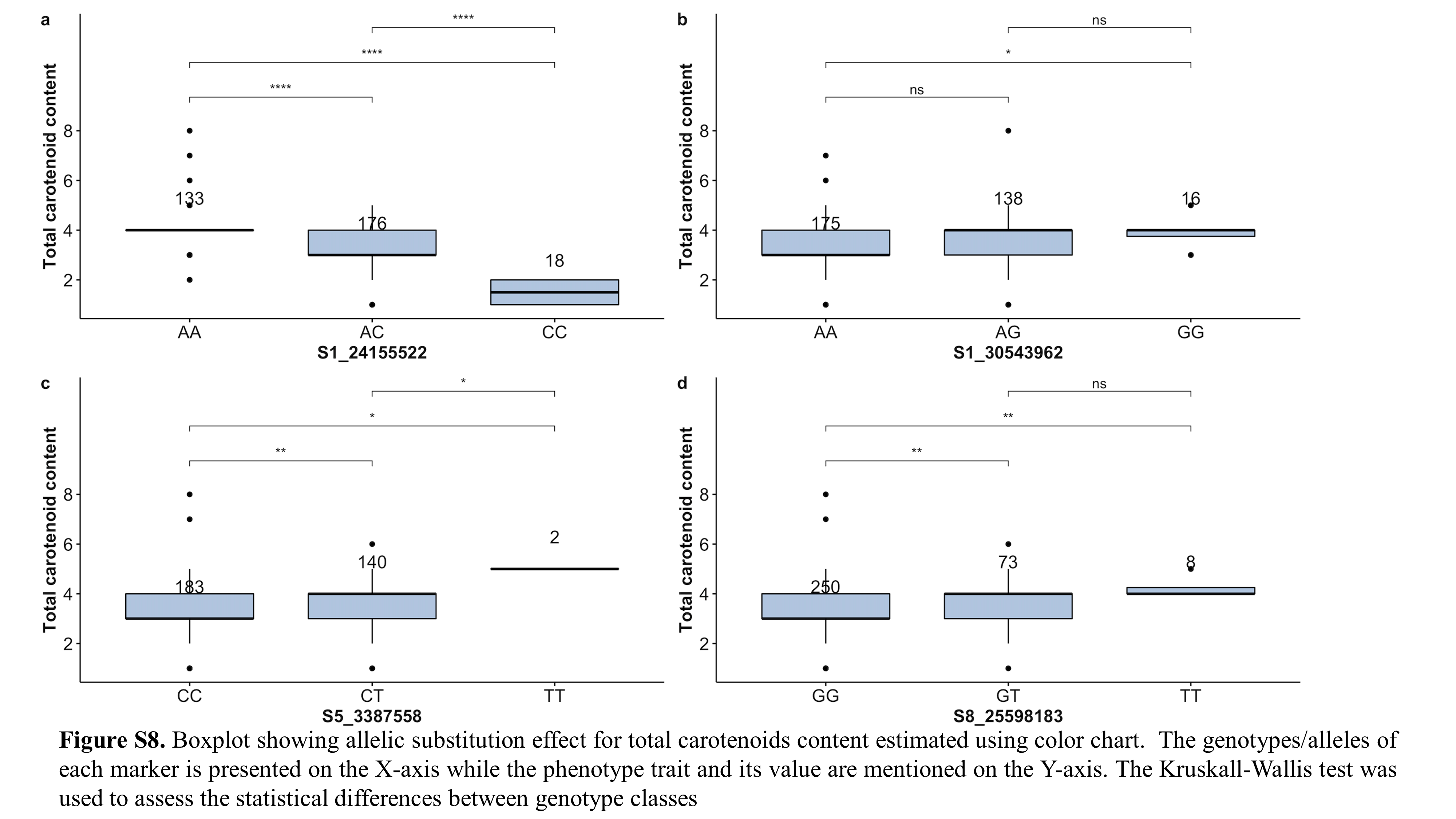

Supplement: Supplementary file 1 [file plants-13-02328-s001.zip › Suppl_all_26June2024/Revised_Supplemental Figures_26June2024/Figure S8.TIF]

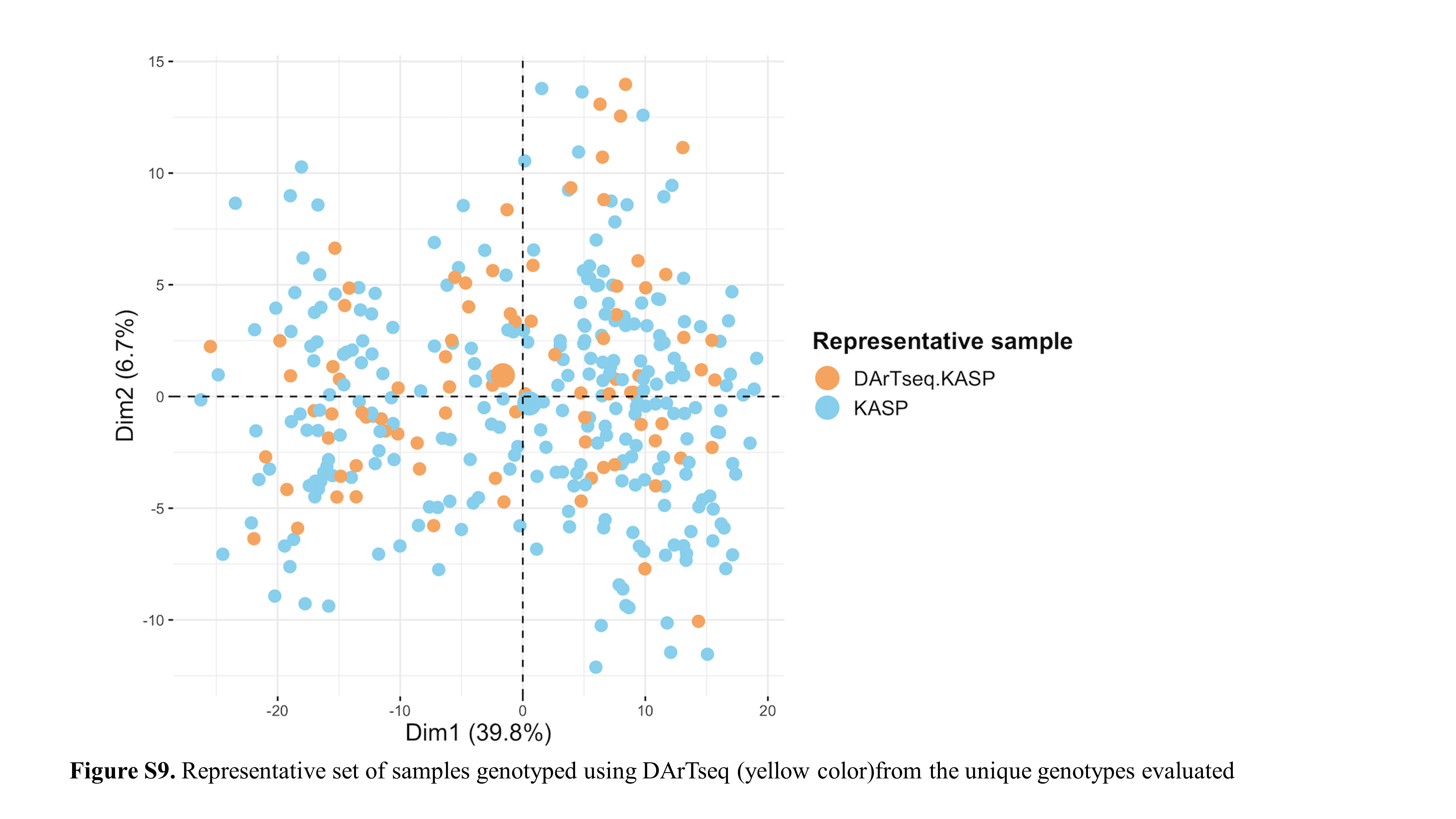

Supplement: Supplementary file 1 [file plants-13-02328-s001.zip › Suppl_all_26June2024/Revised_Supplemental Figures_26June2024/Figure S9.TIF]

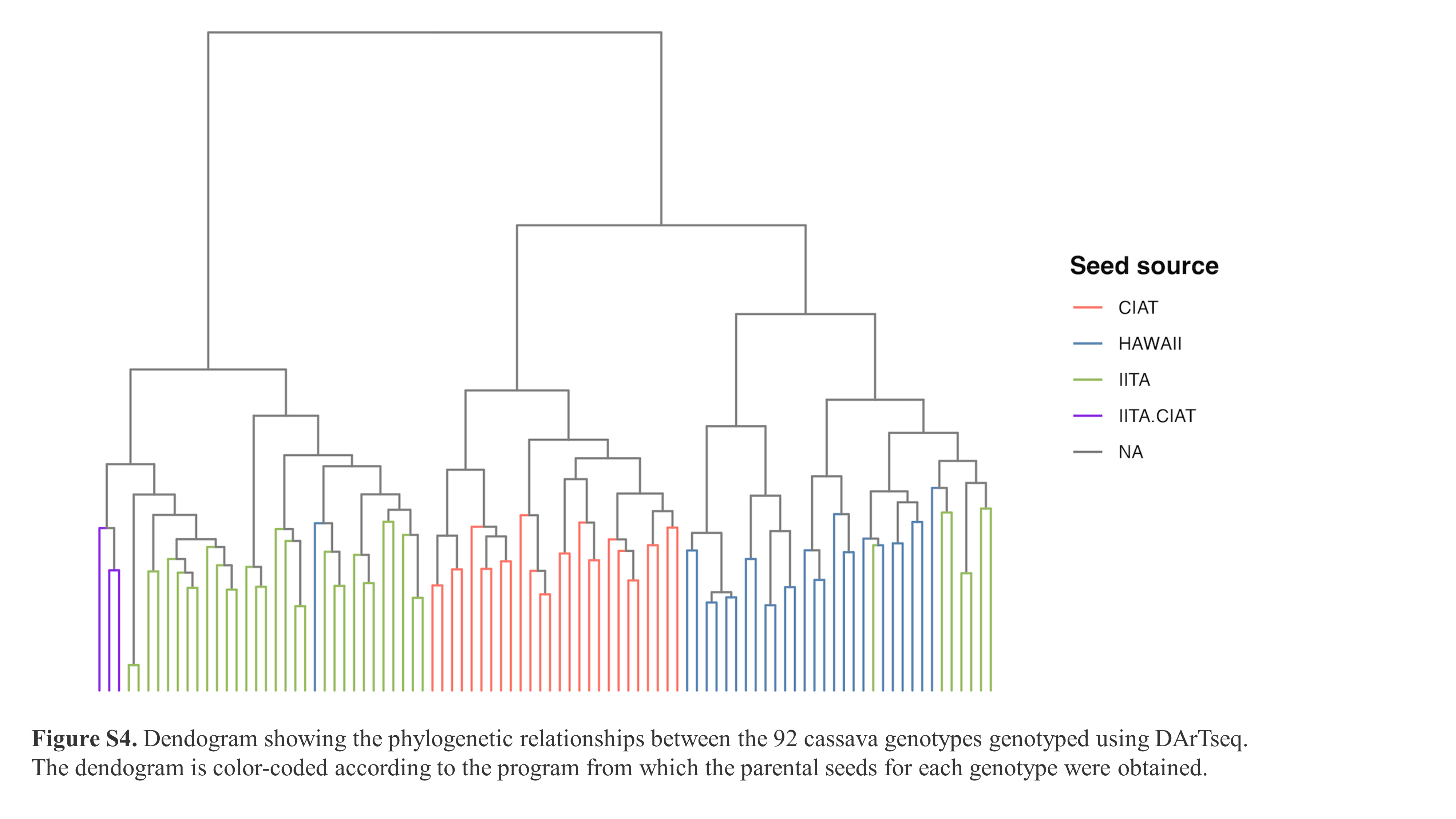

Supplement: Supplementary file 1 [file plants-13-02328-s001.zip › Suppl_all_26June2024/Revised_Supplemental Figures_26June2024/Figure S4.TIF]

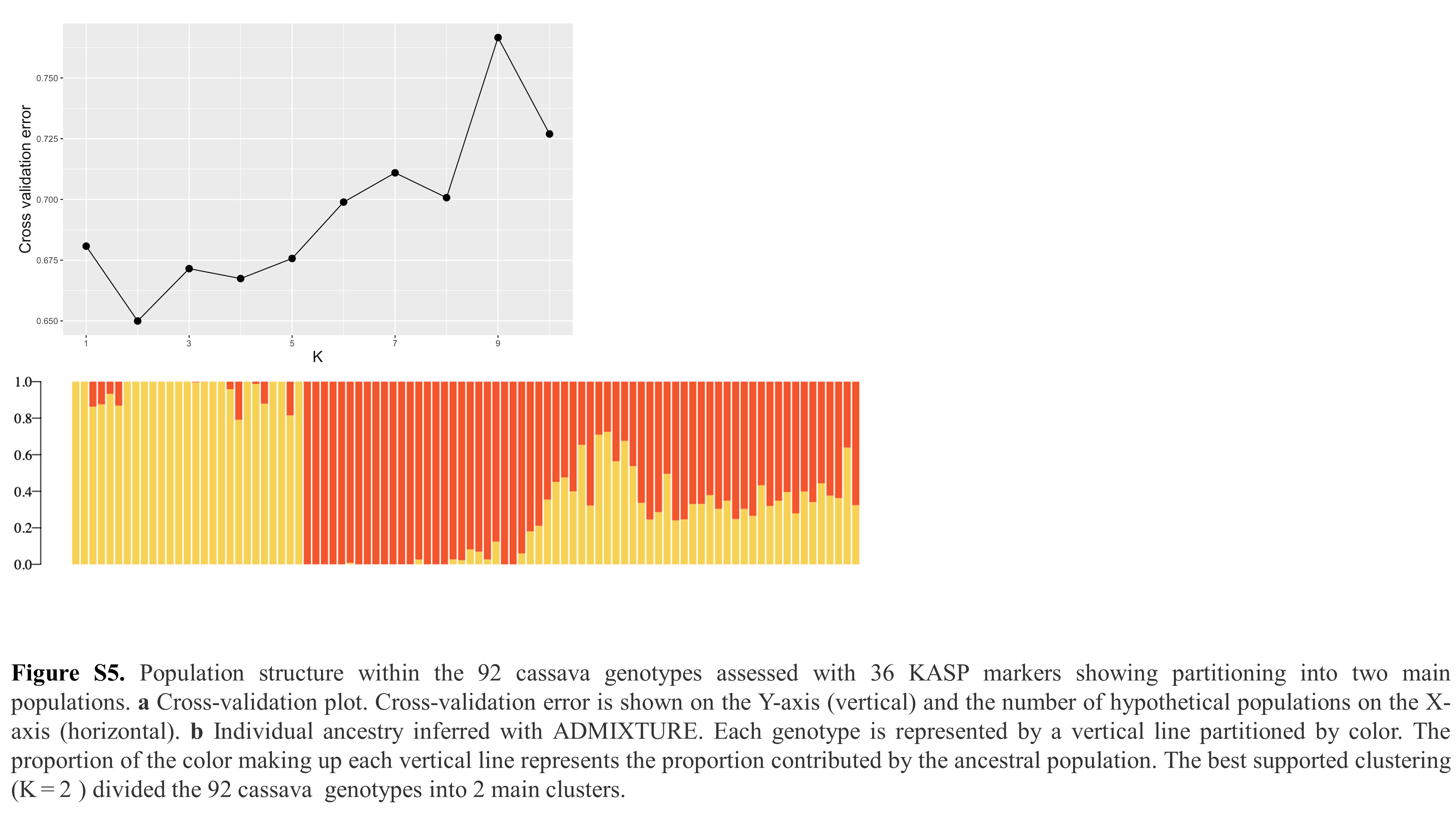

Supplement: Supplementary file 1 [file plants-13-02328-s001.zip › Suppl_all_26June2024/Revised_Supplemental Figures_26June2024/Figure S5.TIF]

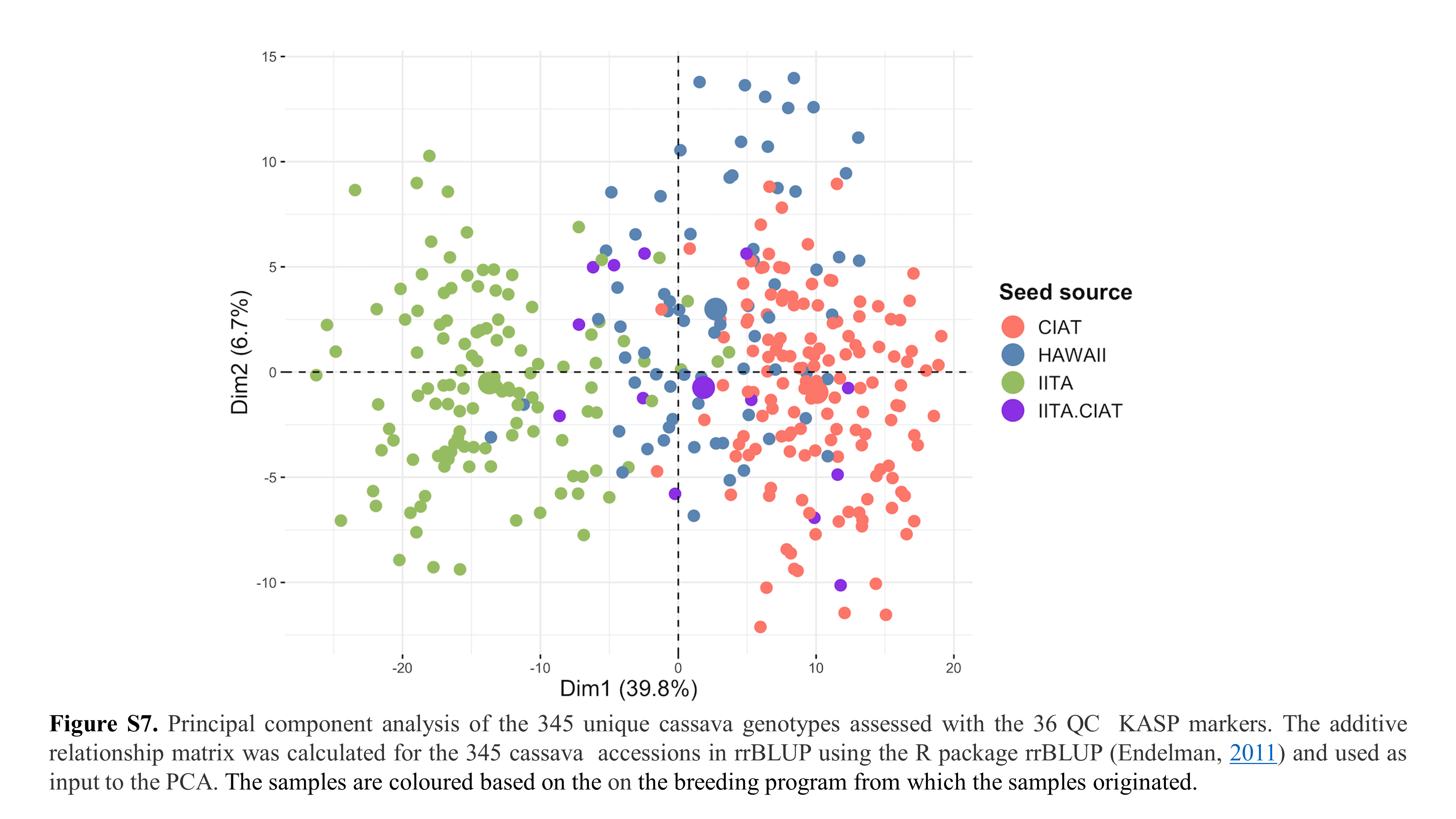

Supplement: Supplementary file 1 [file plants-13-02328-s001.zip › Suppl_all_26June2024/Revised_Supplemental Figures_26June2024/Figure S7.TIF]

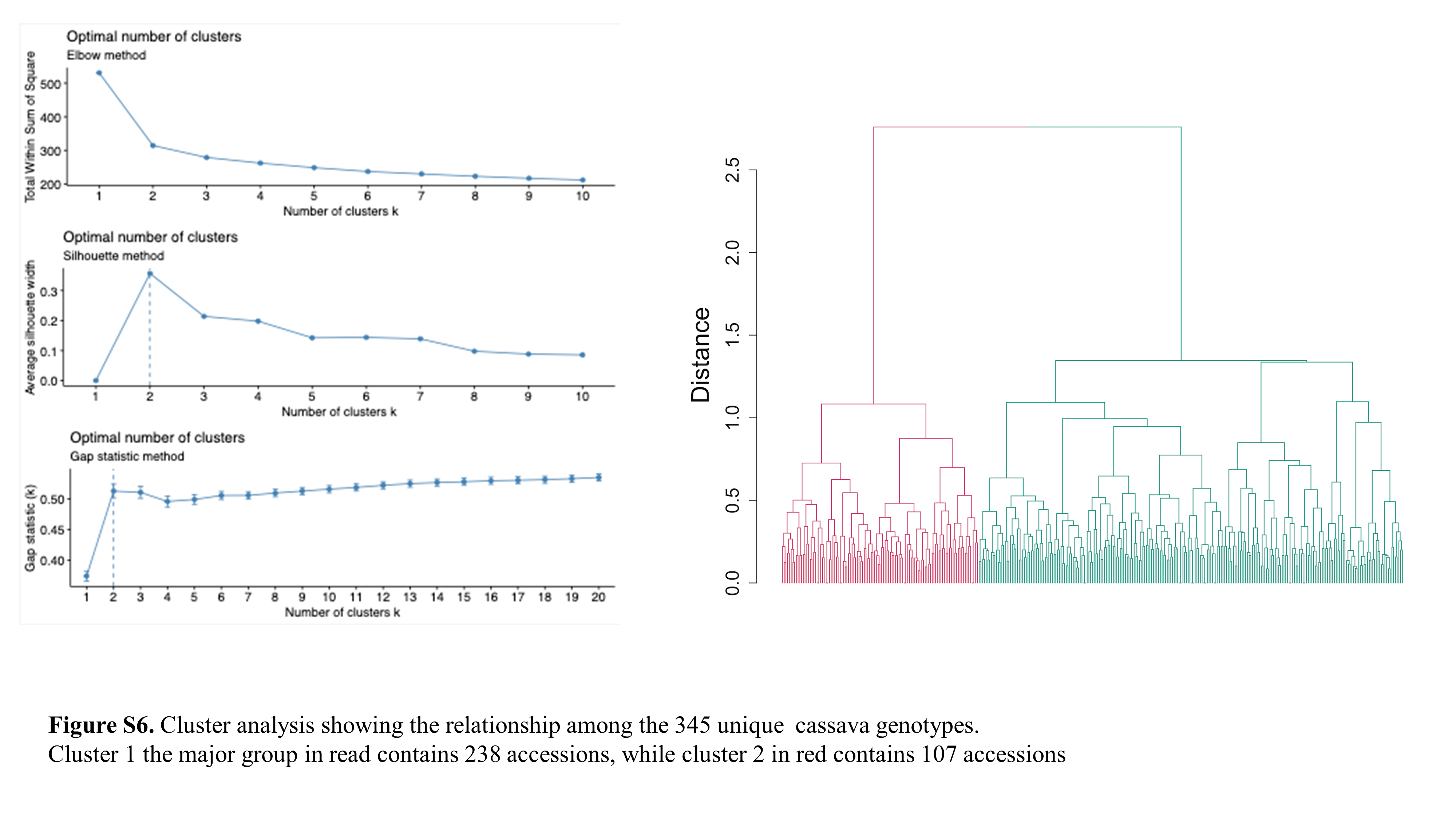

Supplement: Supplementary file 1 [file plants-13-02328-s001.zip › Suppl_all_26June2024/Revised_Supplemental Figures_26June2024/Figure S6.TIF]

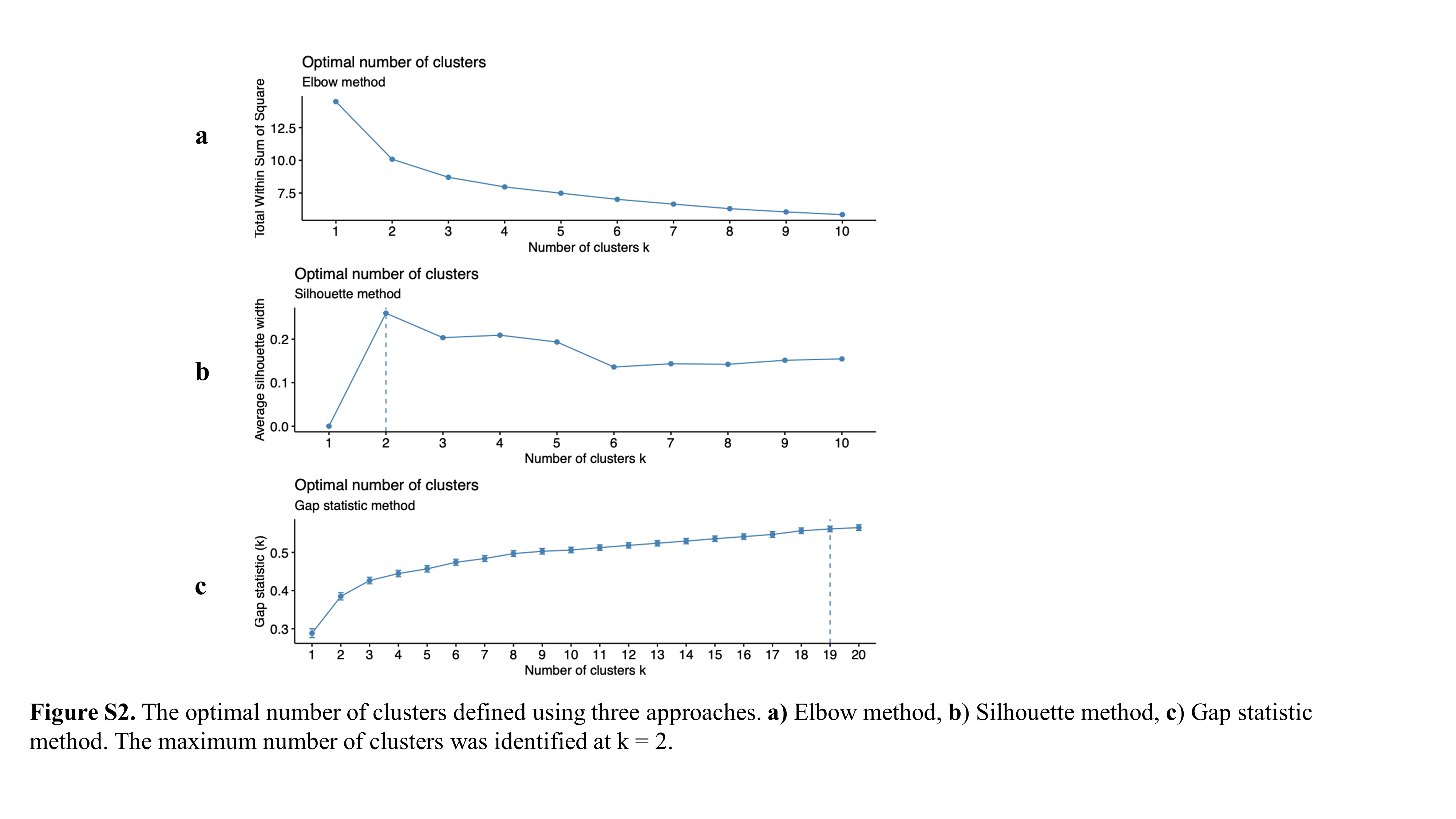

Supplement: Supplementary file 1 [file plants-13-02328-s001.zip › Suppl_all_26June2024/Revised_Supplemental Figures_26June2024/Figure S2.TIF]

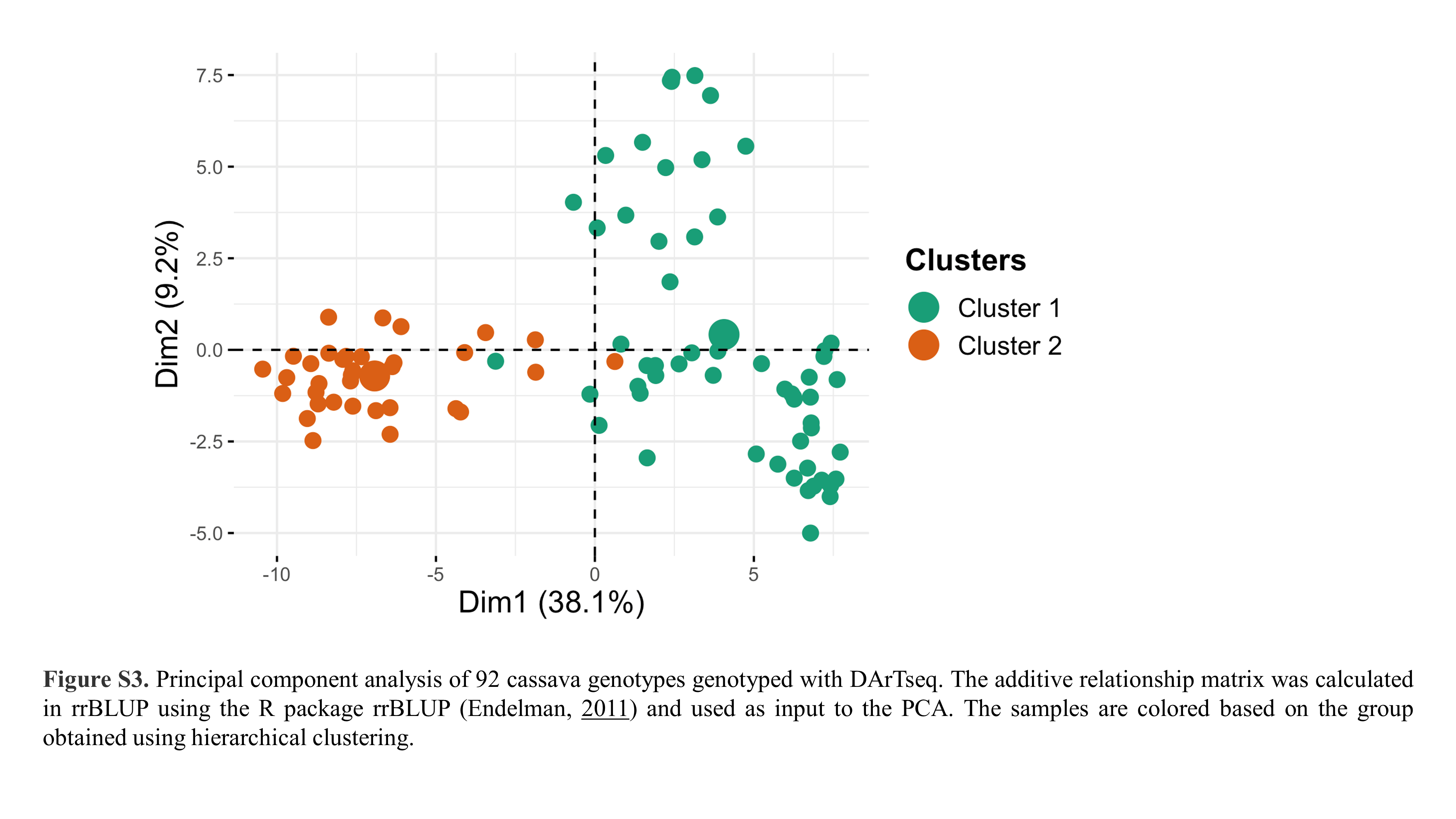

Supplement: Supplementary file 1 [file plants-13-02328-s001.zip › Suppl_all_26June2024/Revised_Supplemental Figures_26June2024/Figure S3.TIF]

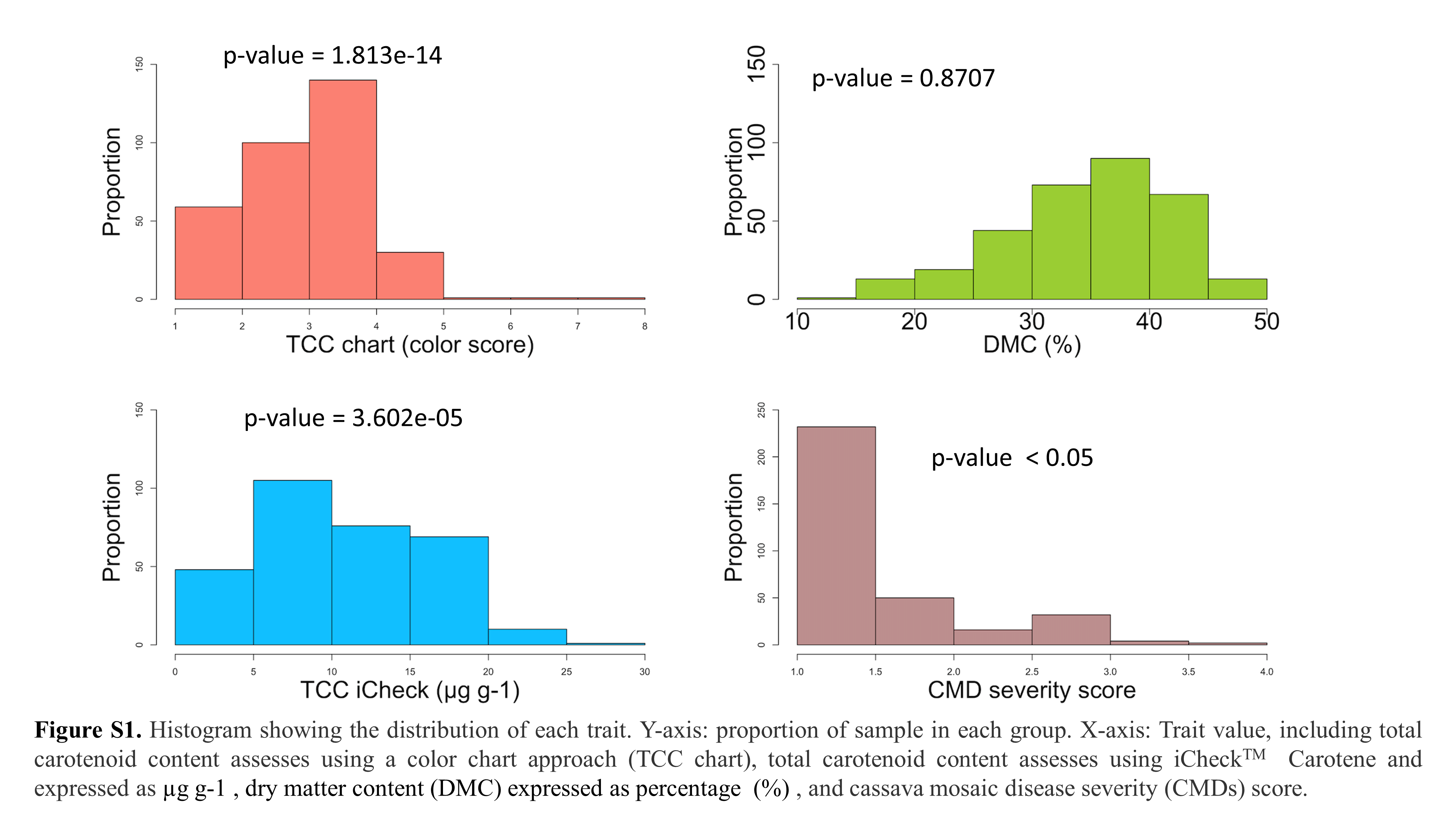

Supplement: Supplementary file 1 [file plants-13-02328-s001.zip › Suppl_all_26June2024/Revised_Supplemental Figures_26June2024/Figure S1.TIF]
